# Genetic aspects of pediatric nephrotic syndrome and anti-nephrin antibodies

**DOI:** 10.1007/s10157-025-02645-4

**Published:** 2025-03-14

**Authors:** Tomoko Horinouchi, Kandai Nozu, Kazumoto Iijima

**Affiliations:** 1 Department of Pediatrics, Kobe University Graduate School of Medicine, Kobe University, 7-5-1 Kusunoki-cho, Chuo-ku, Kobe, 650-0017 Japan; 2 Hyogo Prefectural Kobe Children’s Hospital, Hyogo, Japan; 3 Department of Advanced Pediatric Medicine, Kobe University Graduate School of Medicine, Kobe, Japan

**Keywords:** Anti-nephrin antibodies, Nephrotic syndrome, *NPHS1*, Genome-wide association studies, Steroid-sensitive nephrotic syndrome

## Abstract

Nephrotic syndrome is the most common glomerular disease in children, and various hypotheses regarding its etiology have been proposed, primarily focusing on immune-related mechanisms. Nephrotic syndrome can manifest as a monogenic disease caused by deleterious variants in genes such as *NPHS1*, which encodes nephrin. In steroid-sensitive nephrotic syndrome, HLA class II and immune-related genes have been identified as susceptibility genes. Moreover, *NPHS1* is a susceptibility gene for steroid-sensitive nephrotic syndrome in patients from East Asian populations. Anti-nephrin antibodies have been identified as a significant factor in the pathogenesis of nephrotic syndrome. These discoveries have substantially advanced our understanding of nephrotic syndrome. However, the mechanisms underlying the production of anti-nephrin antibodies and their association with genetic backgrounds have remained unclear and warrant further investigation.

## Introduction

Various etiologies have been proposed for pediatric idiopathic nephrotic syndrome (NS) over time (reviewed in [1]). Clinically, pediatric idiopathic NS is classified into steroid-sensitive NS, which responds to steroids, and steroid-resistant NS, which does not. Patients who achieve complete remission with steroid treatment or additional immunosuppressive therapy are considered to have immunogenic NS, whereas those resistant to all treatments are more likely to have genetic NS. Although a few cases are classified as genetic NS, most pediatric NS cases are classified as immunogenic NS [2]. Since the 1970s, several findings have highlighted the role of T cells in pediatric NS pathogenesis [3, 4]: (1) routine observations show no deposition of immunoglobulins or complement in the glomeruli; (2) immunosuppressive drugs that suppress T cell function (steroids, cyclosporine, and cyclophosphamide) are effective; and (3) remission is achieved in some cases after measles infection, which is associated with decreased T cell function. In the 2010s, the introduction of rituximab, a B-cell-depleting monoclonal antibody, demonstrated high therapeutic efficacy [5, 6], leading to the hypothesis that B cells are also involved in the disease. An increase in B cell subsets, particularly transitional naïve B cells and memory B cells, has been reported in pediatric idiopathic NS, suggesting similarities with autoimmune diseases characterized by impaired immune tolerance [7]. Al-Aubodah et al. analyzed blood samples during the active phase of pediatric idiopathic NS using single-cell RNA sequencing and suggested the activation of extrafollicular B cell responses and the upregulation of the BAFF/APRIL pathway [8]. Extrafollicular responses are considered crucial for the early control of pathogens because of their rapid antibody production and are observed in the early stages of infections or during immune hyperactivation in autoimmune diseases. Additionally, because B cells interact with T cells in antigen presentation [9], it is difficult to separate completely the involvement of T cells and B cells in pediatric NS pathogenesis.

In pediatric patients, NS often relapses or manifests following immune system fluctuations, such as after common viral infections or vaccinations [2]. Furthermore, the prevalence of Epstein–Barr virus (EBV)-DNA is reported to be higher in children with NS onset than in patients in a control group [10]. EBV persists as a latent infection in memory B cells and has been linked to the risk of developing various autoimmune diseases. EBV downregulates CD74, which may be a strategy to evade CD4^+^ Th cell recognition during latent infection in B cells by suppressing HLA class II antigen presentation [11]. Additionally, CD74 downregulation has been reported to lead to the abnormal presentation of self-antigens by HLA class II, which may be recognized as nonself antigens by CD4^+^ Th cells, contributing to the development of systemic lupus erythematosus [12].

Pediatric NS has also been associated with allergic diseases, such as asthma, atopic dermatitis, and hay fever (reviewed in [13]). Elevated serum IgE levels, which can trigger allergic diseases, have been observed in some patients with NS. Allergies are known to arise from Th2 activation due to an imbalance between Th2 and Th1 responses, suggesting a shared mechanism with the onset of NS. Thus, it has been suggested that most pediatric NS is “immunogenic,” as consistent with the findings of genetic investigations and the discovery of anti-nephrin antibodies.

## Insights from genetic studies

### Pediatric NS as a monogenic disease

The elucidation of the pathogenesis of NS has also advanced molecular genetic research on the kidney. In 1998, the group led by Tryggvason cloned *NPHS1,* which encodes a slit diaphragm structural molecule, as the causative gene for Finnish-type congenital NS and named the protein “nephrin” [14]. Nephrin, encoded by *NPHS1*, is an adhesion molecule consisting of 1241 amino acids with an immunoglobulin-like structure. It functions as a bridge between podocyte slit diaphragms. In typical cases of congenital NS caused by *NPHS1* variants, patients present with severe nephrosis from either the prenatal period or shortly after birth. In Finland in particular, truncation-type nephrin variants, which account for 70–80% of nephrin variants in Finnish patients, are often associated with severe phenotypes [14]. In contrast, some patients with single nucleotide variants in nephrin exhibit a milder pathology, resembling minimal change disease, and may present later, at approximately 5–6 years of age, and develop steroid-resistant NS [15, 16]. Additionally, there are reports of patients who experience spontaneous remission or who respond to treatments such as angiotensin-converting enzyme inhibitors or corticosteroids [17, 18].

Various podocyte-related genes have been subsequently revealed to cause genetic NS. Approximately 30% of patients with steroid-resistant NS have a single causative gene associated with podocytes [15, 19, 20].

Furthermore, even in patients with steroid or immunosuppressant-sensitive nephrotic syndrome, *EMP2* variants [21] or genes associated with Rho-like small GTPase activity were identified as causes of NS [22]. However, single causative gene variants are observed in only a few families with steroid-sensitive NS.

### Disease susceptibility genes in the steroid-sensitive nephrotic syndrome-HLA class II region

Genome-wide association studies (GWAS) were first reported globally by RIKEN in the early 2000s and involved the use of 100,000 genetic variants across the entire genome [23]. GWAS is a hypothesis-free method that examines the relationships between genetic variants distributed across the entire human genome and trait information, making it a useful approach for identifying susceptibility genes for common diseases [24].

In 2015, the first hypothesis-free exome-wide study targeting steroid-sensitive NS was conducted, demonstrating for the first time, to our knowledge, that variants in the *HLA-DQA1* region were significantly associated with steroid-sensitive NS [25]. In 2018, a GWAS identified variants significantly associated with the disease in the *HLA-DR/DQ* region through studies conducted on a Japanese cohort and a transethnic GWAS, including populations with European ancestry [26, 27]. HLA-DR and HLA-DQ are MHC class II molecules expressed on antigen-presenting cells. These molecules primarily function in antigen presentation and regulate immune responses [9]. The polymorphisms of these molecules have been implicated in the development of various autoimmune diseases [28–32]. In a GWAS conducted on a Japanese population, the HLA haplotype with the highest risk for disease development [*DRB1*08:02-*DQB1*03:02 (p = 7.01 × 10⁻^11^, Odds ratio [OR] = 3.60)] and the HLA haplotype with the lowest risk [*DRB1*13:02-*DQB1*06:04 (p = 4.18 × 10⁻^12^, OR = 0.10)] were identified [26]. HLA polymorphisms may influence disease susceptibility through differences in peptide-binding affinity [33], interactions with T cells [34], and HLA stability [35]. Moreover, podocytes express MHC class II and exhibit functions similar to immune cells [36]. Further investigations are needed to elucidate how HLA haplotypes contribute to the development of nephrotic syndrome.

### Disease susceptibility genes in steroid-sensitive nephrotic syndrome—outside of the HLA class II region

Most significant associations outside the HLA class II region identified by GWAS involve immune-related molecules, although some associations with kidney-related molecules have also been reported.

Debiec et al. conducted a multiethnic GWAS meta-analysis and reported that, in addition to the aforementioned *HLA-DR/DQ* region, the 3′ untranslated region of *BTNL2* has a significant association with pediatric steroid-sensitive NS (SSNS) [27]. BTNL2 is thought to be a member of the B7 family of costimulatory molecules and is known to modulate T cell activity by suppressing T cell proliferation and cytokine production, promoting regulatory T cell differentiation [37]. *BTNL2* is a susceptibility gene for many autoimmune diseases, including sarcoidosis, ulcerative colitis, Graves’ disease, type 1 diabetes, systemic lupus erythematosus, and rheumatoid arthritis [38]. Dufek et al. analyzed 422 European pediatric SSNS patients and 5,642 controls using GWAS and identified disease susceptibility genes not only in the *HLA-DR/DQ* region but also in the *CALHM6 (FAM26F)* and *PARM1* regions [39]. CALHM6 is highly expressed in lymphocytes and is involved in the immune system [40]. A cis-expression quantitative trait locus (eQTL) has been identified, showing that the minor protective allele is associated with increased expression of CALHM6 [39]. No eQTL has been identified for PARM1 [39], but while the function of PARM1 has not been fully elucidated, it is thought to be potentially involved in endoplasmic reticulum stress [41]. Japanese investigators conducted an expanded GWAS analysis of individuals from a Japanese population in 2020, involving 987 SSNS patients and 3,206 healthy controls. In addition to the *HLA-DR/DQ* region, they identified significant genome-wide variants in the *NPHS1-KIRREL2* and *TNFSF15* regions [42]. As described in the section about monogenic disease, *NPHS1* encodes nephrin [14] and compound heterozygous variants that cause significant changes that typically lead to congenital NS [14]. Interestingly, this study revealed that synonymous and intronic variants of *NPHS1* also influence susceptibility to steroid-sensitive NS. While no difference was observed in the overall mRNA expression levels, in samples with the risk haplotype, the expression of mRNA originating from the risk haplotype was reduced, indicating allele-specific expression [42]. TNFSF15 is involved in apoptosis, cell proliferation, and differentiation into Th1 and Th17 cells [43]. The serum levels of TNFSF15 are elevated in primary biliary cholangitis, inflammatory bowel disease, rheumatoid arthritis, and other inflammatory diseases [44]. Additionally, *TNFSF15* is a susceptibility gene for various immune-related diseases, and rs4979462, one of the single nucleotide polymorphisms (SNPs) significantly associated with SSNS increases the expression of the endogenous TNFSF15 protein and mRNA [45].

### Multipopulation GWAS meta-analyses

In rare diseases such as SSNS, multipopulation GWAS meta-analyses are valuable for increasing sample sizes. Furthermore, multipopulation GWAS meta-analyses can improve the generalizability and reproducibility of disease susceptibility genes and provide a comprehensive understanding of genetic diversity. For pediatric SSNS, multipopulation GWAS meta-analyses, conditional analyses, and population-specific GWASs were conducted, with the results reported in 2023 [46]. This study included 2,240 pediatric SSNS patients from 6 ethnic groups worldwide (including 987 Japanese individuals) and 36,023 healthy controls from the corresponding ethnic groups. In addition to the 4 previously known susceptibility genes, including HLA class II, 7 new disease susceptibility genes were identified. Evaluation of eQTLs in immune cells and kidney tissue for genome-wide significant variants revealed that several variants act as eQTLs in immune cells but not in kidney tissue.

*NPHS1* is a disease susceptibility gene exclusive to East Asian populations [42]. Additionally, variants in many gene regions overlap with open chromatin regions in both immune cells and kidney cells [46]. Downie et al. conducted a GWAS involving 420 Sri Lankan SSNS patients and 2,239 healthy controls. A meta-analysis with the European GWAS [39] identified a genome-wide significant variant in the *AHI1* region and the *HLA-DR/DQ* region [47]. *AHI1* is known to be involved in ciliary protein transport and immune dysregulation, and rare variants in this gene contribute to the development of Joubert syndrome, a ciliopathy [48, 49]. eQTL analysis of the lead SNP, rs2746432, revealed that the risk allele of this variant is associated with decreased *AHI1* expression [47].

### Post-GWAS analysis

#### Polygenic risk score

One of the analyses performed after GWAS is the creation of a polygenic risk score (PRS). Disease susceptibility variants identified through GWASs have small effect sizes, meaning that their individual impact on disease is limited, making it challenging to predict disease onset based on a single variant. To address this, the PRS was proposed based on the “polygenic model,” a hypothesis that numerous variants across the genome collectively and additively increase disease risk. The PRS is calculated as the sum of the product of each variant’s effect size and the number of risk alleles carried by an individual [50]. For SSNS, in the multipopulation GWAS meta-analyses mentioned earlier, a PRS was developed [46]. Using summary statistics from 1,974 cases and 20,039 controls across European, East Asian, African, and South Asian populations, a multipopulation PRS was created. Calculating PRS across diverse ethnicities is typically challenging owing to differences in allele frequencies, effect sizes, and linkage disequilibrium regions among populations. However, this study employed PRS-CSx [51], an analytical method that accounts for these factors.

Because this PRS was developed with parameter adjustment based on European data, it cannot be directly applied to other populations. Therefore, developing a PRS tailored explicitly for each population is warranted.

### Anti-nephrin antibodies in NS

#### Discovery of anti-nephrin antibodies

Watts et al. reported that circulating anti-nephrin antibodies were detected by ELISA in 18 of 62 (29%) patients with minimal change NS (MCNS) and that their titers were strongly correlated with disease activity [52]. They also demonstrated the colocalization of IgG and nephrin in renal biopsies during active disease phases, suggesting that autoimmunity against nephrin (anti-nephrin antibodies) is likely one of the pathogenic mechanisms of MCNS. Furthermore, anti-nephrin antibodies have also been reported to play a role in the recurrence of primary focal segmental glomerulosclerosis (FSGS) after transplantation [53, 54]. Shirai et al. reported that all 11 cases of recurrent FSGS (rFSGS) following kidney transplantation were positive for anti-nephrin antibodies and demonstrated punctate IgG deposits colocalizing with nephrin in kidney pathology [54]. Batal et al. reported that among patients who exhibited rFSGS, 8 of 21 (38%) tested positive for anti-nephrin antibodies in pretransplant serum, whereas all 17 patients who did not experience recurrence tested negative [55]. In a larger cohort, Hengel et al. utilized a quantitative immunoprecipitation–ELISA hybrid assay to measure anti-nephrin antibodies in 539 patients (357 adults and 182 children) and 117 controls [56]. Among adults, anti-nephrin autoantibodies were detected in 46 of 105 (44%) patients with MCNS and 7 of 74 (9%) patients with primary FSGS, while they were rarely found in other diseases. In children with idiopathic NS, anti-nephrin autoantibodies were detected in 94 of 182 (52%) patients. In a subgroup of patients with idiopathic NS who had not received immunosuppressive therapy and exhibited high disease activity, the prevalence of anti-nephrin autoantibodies was high, with 35 of 39 (90%) patients testing positive. Additionally, anti-nephrin antibody levels are correlated with disease activity [56]. Horinouchi et al., in collaboration with Watts et al., analyzed anti-nephrin antibodies in paired plasma samples collected during the onset (active phase) and after steroid monotherapy in 14 Japanese pediatric patients with idiopathic NS [57]. Among the 14 patients, 13 achieved complete remission after 4 weeks of steroid therapy, while the remaining patient achieved partial remission. Circulating anti-nephrin antibodies were detected in 7 of 14 (50%) patients during the active phase. In all patients, anti-nephrin antibody titers significantly decreased in parallel with clinical improvement following treatment [57].

The sensitivity and specificity of anti-nephrin antibodies may vary depending on the assay. A situation in which anti-nephrin antibodies can be reliably measured in any laboratory needs to be established, along with evaluations of their positivity rates, the timing of seroconversion and seroreversion, and associations with patient characteristics across various cohorts.

That nephrotic syndrome is induced by circulating antibodies is consistent with previous reports that nephrotic syndrome can be caused by circulating factors [58]. Evidence suggesting the involvement of circulating factors includes, for example, that approximately 30% of patients with FSGS develop massive proteinuria within hours to days, followed by FSGS histological lesions, after receiving a kidney transplant from a donor without the disease [59]. In addition, early plasma exchange is sometimes effective, and preemptive plasmapheresis reduces the risk of recurrence [60]. Furthermore, perfusion of isolated rat glomeruli with plasma from patients with FSGS increased glomerular capillary permeability to albumin compared with perfusion by normal control plasma [61]. The presence of circulating anti-nephrin antibodies may explain these findings. Anti-nephrin antibodies can induce nephrotic syndrome in mice [56]. When mice were actively immunized with the extracellular domain of mouse nephrin, anti-mouse nephrin autoantibodies were generated, and nephrotic syndrome developed 3 weeks later. Histologically, the findings were consistent with a MCNS-like phenotype, suggesting that anti-nephrin antibodies can induce MCNS [56].

### Autoantibodies other than anti-nephrin antibodies

Raglianti et al. reported that antibodies against slit diaphragm molecules other than nephrin may also exist [62]. Among the pediatric patients who underwent renal biopsy, 30 with MCNS and 32 with FSGS were analyzed. Among these patients, 45 presented with clinical features of steroid-resistant NS. Among these 45 cases, anti-slit diaphragm antibodies were detected in 18 cases: 10 of 30 MCNS cases and 8 of 32 FSGS cases. In 14 of the 18 anti-slit diaphragm antibody-positive cases, nephrin and IgG were colocalized, suggesting the presence of anti-nephrin antibodies. However, in the remaining 4 patients, the colocalization of nephrin and IgG was inconsistent, indicating the possible presence of anti-slit diaphragm antibodies that are not directed against nephrin. Raglianti et al. identified anti-podocin and anti-NEPH1 autoantibodies as anti-slit diaphragm antibodies, broadening the spectrum of autoimmune podocytopathies beyond anti-nephrin antibodies [63]. Further investigations are needed to clarify the clinical importance of these autoantibodies and their potential utility in clinical practice.

### Future perspectives

The identification of monogenic causative genes, such as *NPHS1*, and GWASs aimed at uncovering susceptibility genes have significantly advanced our understanding of the pathophysiology of pediatric NS. Furthermore, the recent discovery of anti-nephrin antibodies supports the concept of NS driven by immunological mechanisms and represents a groundbreaking development in this field.

However, there remains a lack of data about why anti-nephrin antibodies are produced and how they are associated with genetic characteristics. It is hoped that future research will elucidate the mechanisms underlying the production of anti-nephrin antibodies, their relationship with disease activity, treatment responsiveness, and genetic characteristics, as well as the identification of other autoantibodies and their associations with the pathophysiology of the disease.

## Data Availability

Data sharing is not applicable to this article as no new data were created or analyzed in this review.
